# Exosome-Based Cell-Cell Communication in the Tumor Microenvironment

**DOI:** 10.3389/fcell.2018.00018

**Published:** 2018-02-20

**Authors:** Joana Maia, Sergio Caja, Maria Carolina Strano Moraes, Nuno Couto, Bruno Costa-Silva

**Affiliations:** Systems Oncology Group, Champalimaud Research, Champalimaud Foundation, Lisbon, Portugal

**Keywords:** exosomes, cancer, tumor microenvironment, extracellular vesicles (EVs), cell-cell communication

## Abstract

Tumors are not isolated entities, but complex systemic networks involving cell-cell communication between transformed and non-transformed cells. The milieu created by tumor-associated cells may either support or halt tumor progression. In addition to cell-cell contact, cells communicate through secreted factors via a highly complex system involving characteristics such as ligand concentration, receptor expression and integration of diverse signaling pathways. Of these, extracellular vesicles, such as exosomes, are emerging as novel cell-cell communication mediators in physiological and pathological scenarios. Exosomes, membrane vesicles of endocytic origin released by all cells (both healthy and diseased), ranging in size from 30 to 150 nm, transport all the main biomolecules, including lipids, proteins, DNAs, messenger RNAs and microRNA, and perform intercellular transfer of components, locally and systemically. By acting not only in tumor cells, but also in tumor-associated cells such as fibroblasts, endothelium, leukocytes and progenitor cells, tumor- and non-tumor cells-derived exosomes have emerged as new players in tumor growth and invasion, tumor-associated angiogenesis, tissue inflammation and immunologic remodeling. In addition, due to their property of carrying molecules from their cell of origin to the peripheral circulation, exosomes have been increasingly studied as sources of tumor biomarkers in liquid biopsies. Here we review the current literature on the participation of exosomes in the communication between tumor and tumor-associated cells, highlighting the role of this process in the setup of tumor microenvironments that modulate tumor initiation and metastasis.

## Introduction

The tumor microenvironment is anything but simple. Be it the primary or the metastatic tumor, its complex and heterogeneous microenvironment is comprised of a network of both cellular and acellular constituents. While the former consists of tumor cells and diverse non-transformed cells, such as cancer-associated fibroblasts, macrophages, and endothelial cells, the latter is formed by secreted factors and components of the extracellular matrix (ECM). In its intricacy, the tumor microenvironment has emerged to be a key modulator of tumor progression by providing inhibitory or stimulatory growth signals (Bissell and Hines, 2011).

Interactions amongst neighboring cells in the primary tumor are essential for tumor growth and development, and besides direct cell-cell contact, intercellular communication also happens through a complex system involving secreted factors. Besides local cell-to-cell communication, secreted factors play a key role in the interaction amongst cells located far apart from each other (Becker et al., 2016; Fu et al., 2016; Kalluri, 2016; Peinado et al., 2017). In the group of secreted factors, we will here focus on the roles of exosomes in setting up and modifying tumor microenvironments.

**Figure 1:**
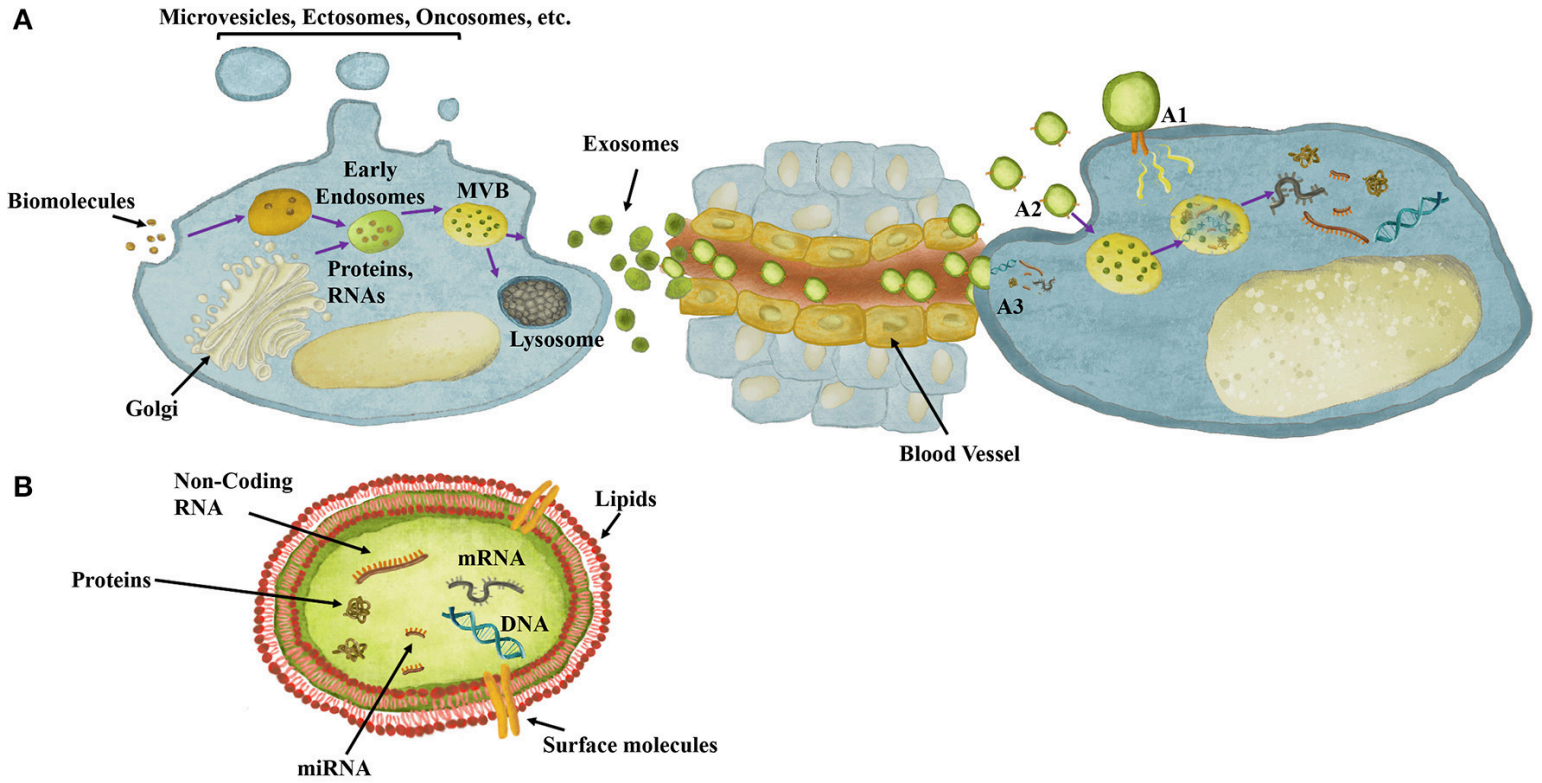
Exosomes role in cell-cell communication and their content. **(A)** Exosomes are extracellular vesicles composed of biomolecules derived from, for instance, Golgi and endocytosis, which are processed through endosomal compartments into multivesicular bodies (MVB). MVBs can either fuse with lysosomes for degradation or with the plasma membrane for release of exosomes to the extracellular milieu. Once released, exosomes can act both locally or travel through the circulation reaching distant sites. Exosomes mediate cell-cell communication though different mechanisms. **(A1)** Exosomes may dock at the plasma membrane of the target cell and activate intracellular signaling by ligand-receptor interaction. **(A2)** Exosomes may be endocytosed by phagocytosis, micropinocytosis or receptor-/raft-mediated endocytosis, and fuse with the delimiting membrane of an endocytic compartment, releasing their content into the cytoplasm of the recipient cells. **(A3)** Exosomes may be directly taken up by membrane fusion, releasing their content into the cytoplasm. **(B)** Exosomes structure, involving a double-layered lipid membrane vesicle containing every basic cellular biomolecule, including Proteins, DNA, mRNA and miRNA.

The study of exosomes and other extracellular vesicles (EVs) is a relatively new field of research that picked up steam in the last couple of decades. The first indication of the existence of EVs came in 1946, when Chargaff and West showed that a platelet-free plasma fraction maintained clotting properties, which was diminished after a high-speed ultracentrifugation that pelleted a particulate fraction with coagulatory activity (Chargaff and West, 1946). After two more studies in the 1950's on the role of alike fractions in promotion of coagulation (Hougie, 1955; O'Brien, 1955), in 1967 Peter Wolf published his seminal work showing by electron microscopy the “coagulant material in minute particulate form” pelleted by ultracentrifugation. Wolf called these lipid rich particles originated from platelets “platelet-dust,” and also suggested that these lipid particles might be normally liberated in circulating blood (Wolf, 1967). Decades later, platelet-derived microparticles were in fact shown to be the most abundant extracellular vesicle population in peripheral blood (Zmigrodzka et al., 2016). In the early 1980's, two articles were published reporting that these particles could also be produced by tumor cells (Dvorak et al., 1981, 1983). Later, in 1987, while studying reticulocyte maturation, Johnstone and colleagues introduced for the first time the term “exosomes” to describe these particles (Johnstone et al., 1987). By then, exosomes were commonly seen as “trashbags” for excreting cellular components, but in 1998 Sebastian Amigorena's group proposed a role for exosomes in the communication between cells of the immune system, thus making exosome's debut as a mediator of cell-cell communication (Zitvogel et al., 1998). Few years after, in 2001, it was shown that platelet-derived EV could transfer antigens like CD41 from platelets to the cell membranes of CD34+ hematopoietic stem-progenitor cells, demonstrating the ability of EVs to horizontally transfer information (Janowska-Wieczorek et al., 2001; Figure 1A). Platelet-derived EV were also shown to induce tumor chemotaxis, proliferation, invasion and expression of angiogenic factors, contributing to the formation of distant metastasis (Janowska-Wieczorek et al., 2005, 2006; Toth et al., 2008; Dashevsky et al., 2009). In addition, they have been shown promote thrombus formation and contribute to metastasis (Falanga et al., 2003), being plasmatic levels of these EVs associated aggressiveness, prognosis and survival of oncologic patients (Kim et al., 2003; Helley et al., 2009; Italiano et al., 2010; Voloshin et al., 2014).

Being still an immature scientific field, EVs' research still faces several basic challenges. The nomenclature of distinct types of EVs, the lack of good established markers and the technical difficulties and heterogeneity of isolation protocols, for instance, have in the last few years been subject of extensive evaluations and debates. In an attempt to improve standardization in the field, the International Society for Extracellular Vesicles recently released guidelines for the analysis of EVs and the reporting of the results (Lotvall et al., 2014), a standardization initiative followed by others, such as the EV-TRACK Consortium (EV-TRACK Consortium et al., 2017). EVs can be classified according to size and cellular origin (endosome- or plasma membrane-derived). Differently from larger EVs, which are released directly from the plasma membrane of both living and dying cells, such as microvesicles and apoptotic bodies, and from vesicular artifacts composed of disrupted membrane fragments spontaneously released by cells undergoing necrosis, known as microsomes (Witwer et al., 2013), exosomes are small EVs, ranging in size from 30 to 150 nm, with a multivesicular endosomal origin actively secreted by all cell types upon fusion of Multivesicular Bodies with the plasma membrane (Becker et al., 2016; Kalluri, 2016). However, most present-day EV's isolation protocols do not discriminate EVs according to their origin, and only recently Kowal and colleagues showed that only the subpopulation bearing the three Tetraspanins CD9, CD63, and CD81 corresponds to endosome-derived exosomes. Another source of confusion is the fact that current isolation protocols provide enriched fractions of EVs, but not pure fractions (Kowal et al., 2016). Thus, many studies to date consider small vesicles as exosomes regardless of cellular origin, and for the purposes of this review we do the same. Also, various nomenclatures have been used to describe small EVs over the years, and for the sake of clarity and according to the protocols the authors used for EV isolation we will call these small EVs exosomes, even when authors use other terms to refer to these vesicles.

Although not yet completely characterized, the process of exosomes biogenesis and release to the extracellular microenvironment relies on several energy-dependent active steps mediated by, for instance, SNAREs and Rabs and Ras GTPases (Pfeffer, 2007). During this process, exosomes are packed with proteins, lipids, DNAs, messenger RNAs (mRNAs), microRNAs (miRNA) and other non-coding RNAs (Becker et al., 2016; Figure 1B). As in other scenarios of cellular stress, including hypoxia and ER stress, exosomes production and composition has been described as markedly affected in oncologic settings, where the role of exosomes as mediators of cell-to-cell communication has been broadly studied. Exosomes' concentration is frequently higher in the blood of cancer patients when compared with healthy control human blood, and the cargo of exosomes can change according to the patient's health status (Kalluri, 2016). Here we will discuss the anti- and pro-tumorigenic effects of tumor and non-tumor cells-derived exosomes in tumorigenic and metastatic processes, focusing on how exosomes mediate interactions amongst tumor cells, leukocytes, fibroblasts, tumor-associated vasculature, and stem/progenitor cells relevant to the tumor microenvironment.

## Exosomes in tumor-tumor communication

The proliferation of tumor cells, a process indispensable for cancer progression, relies on soluble growth factors. As mentioned above, cells also convey information to the microenvironment through molecules packed in exosomes and other EVs through complex signaling networks that are only beginning to be unveiled. Studies involving several distinct cancer cells showed that tumor-derived exosomes can induce tumor cell proliferation. For example, an autocrine induction of cellular proliferation was observed in chronic myeloid leukemia (Raimondo et al., 2015) and in human gastric cancer (Qu et al., 2009; Pan et al., 2017) through, for instance, the phosphatidylinositol 3-kinase/protein kinase B (PI3K/AKT) and MAPK/ERK signaling pathways (Qu et al., 2009) and transference of a long noncoding RNA (Pan et al., 2017). Still in gastric cancers, the signaling for tumor cell proliferation through MAPK can be mediated by exosomal CD97 (Li C. et al., 2015). In human bladder cancer, induction of cell proliferation was observed when the human bladder cancer cells T24 and 5637 were treated with T24 tumor cell-derived exosomes, in a mechanism also mediated by activation of the Akt and ERK pathways (Yang et al., 2013). In addition, glioblastoma-derived exosomes were shown to induce proliferation of the human glioma U87 cell line (Skog et al., 2008) in a mechanism dependent of the CLIC1 protein (Setti et al., 2015). In a narrower context related to prostate cancer treatment, namely the diminished availability of androgens caused by androgen-deprivation therapy, prostate cancer LNCaP cells cultured in the presence of androgens secrete exosomes enriched in CD9, which in turn induce the proliferation of androgen-deprived LNCaP cells (Soekmadji et al., 2016). Another example involves the promotion of *in vivo* growth of murine melanomas by systemic treatment of mice with melanoma-derived exosomes, which a ccelerated growth and inhibited apoptosis of melanoma tumors *in vivo* (Matsumoto et al., 2017).

In addition to the effects on cell proliferation, tumor-derived exosomes can also modify the migratory status of recipient malignant cells. Nasopharyngeal carcinoma-derived exosomes carrying Epithelial to Mesenchymal transition (EMT)-inducing signals, including TGF-β, Hypoxia-Inducible Factor 1 alpha (HIF1α) (Aga et al., 2014), Matrix Metalloproteinases (MMPs) (You et al., 2015), Notch1, LMP1 Casein Kinase II and Annexin A2 (Yoshizaki et al., 2013; Jeppesen et al., 2014; Kruger et al., 2014; Ung et al., 2014; Cha et al., 2015), were shown to enhance the migratory capacity of the tumor recipient cells. Another example involves exosomes derived from hypoxic prostate cancer cells, which induced increased invasiveness and motility of naïve human prostate cancer cells (Ramteke et al., 2015).

In addition to several works reporting their pro-tumorigenic effects, exosomes were also shown to play a role in tumor-tumor communication by transferring chemoresistance. Since Corcoran and colleagues reported that exosomes can transfer Docetaxel resistance in prostate cancer (Corcoran et al., 2012), similar phenomena have been described in distinct tumor contexts, such as in lung, breast and liver cancers (Takahashi et al., 2014; Xiao et al., 2014; Kong et al., 2015). Indeed, in lung cancer the transfer of Cisplatin resistance is mediated by production of exosomes containing low levels of miRNA miR-100-5p by donor resistant cells, which in turn leads to an increased expression of the mammalian target of Rapamycin (mTOR) protein and chemoresistance in the recipient cells (Qin et al., 2017). In breast cancer, miRNA packed in exosomes from drug-resistant cells can modify the expression of specific target genes, including Sprouty2 (targeted by miR-23a), PTEN (targeted by miR-222), APC4 (targeted by miR-452) and p27 (targeted by miR-24), modulating chemoresistance in recipient cells that incorporate these exosomes (Chen et al., 2014a; Mao et al., 2016). In fact, exosomal miR-222 plays a key role in this process (Chen et al., 2014b; Yu et al., 2016), as the silencing of miR-221/222 prevents the transmission of resistance (Wei et al., 2014). Besides miRNAs, the transfer of exosomal mRNAs that encode proteins that confer drug resistance may lead to chemoresistance in the recipient cell. GSTP1 exosomal mRNA from breast cancer cells resistant to Adriamycin, for instance, confer resistance to previously sensitive cells. Importantly, identification of GSTP1 in circulating exosomes from peripheral blood of patients was correlated with worst prognosis in breast cancer patients treated with Adriamycin (Yang et al., 2017).

## Exosomes in tumor-fibroblast communication

An ideal metabolic and physiological environment for tumor growth requires a supportive stroma. Fibroblasts are the most abundant cells in the majority of solid tissues, participating in responses to environmental cues and constituting a frequent target of tumor-derived signals (Olumi et al., 1999; Orimo et al., 2005; Hu et al., 2015). Amongst these signals, exosomes produced by tumor cells have been described as important modulators of the activation status of fibroblasts and to play a major role in the setup of tumor microenvironments (Table 1). One of the factors involved in the activation of these cells, frequently named Cancer-Associated Fibroblasts (CAFs), is Transforming Growth Factor beta (TGF-β) (Tomasek et al., 2002), which can be carried to the extracellular milieu by exosomes and induce differentiation of CAFs (Webber et al., 2010, 2015). In addition, prostate cancer-derived exosomes containing miR-100, −21, and −139, were shown to induce RANKL and Metalloproteinases expression in CAFs, playing a potential role in prostate cancer progression and metastasis (Sanchez et al., 2016). Furthermore, under hypoxic conditions, prostate cancer cells release exosomes containing nearly three times more proteins than those in normoxic conditions, which induce activation of CAFs (Ramteke et al., 2015), and have been associated with the promotion of EMT, stemness, and angiogenesis by prostate cancer cells (Giannoni et al., 2010; Fiaschi et al., 2013). Tumor-derived exosomes were also described as regulators of metabolism in the tumor microenvironment, as breast cancer tumors could suppress glucose uptake by non-tumor cells, including lung fibroblasts, via secretion of exosomes containing miR-122, increasing glucose availability and facilitating metastasis (Fong et al., 2015).

**Table 1:** Exosomal-mediated phenotypes associate with cancer progression.

**Process**	**Exosomal factor**	**Effect**	**References**
**TUMOR-DERIVED EXOSOMAL FACTORS**			
Cancer progression and metastasis	TGF-β	Differentiation of fibroblast into CAF	Webber et al., 2010, 2015
	miR-100, −21, −139	ECM remodeling and modulation of cell migration	Sanchez et al., 2016
	TGF-β2, TNF 1α, IL-6, TSG101, Akt, ILK1, β-Catenin	Activation of prostate CAF by promotion of EMT, stemness and angiogenesis	Giannoni et al., 2010; Fiaschi et al., 2013; Ramteke et al., 2015
	Neuroblast Differenciation-Associated protein (AHNAK)	Differentiation of fibroblast into CAF	Silva et al., 2016
Metabolic Environment	miR-122	Suppression of glucose uptake	Fong et al., 2015
Establishment of Pre-metastatic niches	hTERT mRNA	Increased fibroblast proliferation and lifespan	Gutkin et al., 2016
	CD151, Tetraspanin 8	Fibrosis and ECM remodeling	Yue et al., 2015
	Integrin α6β1, α6β4	Lung-pre-metastatic niche formation mediated by S100A4, A6, A10, A11, A13, and A16	Hoshino et al., 2015
**FIBROBLAST-DERIVED EXOSOMAL FACTORS**			
Cancer progression and/or metastasis	EphA2	Induction of proliferation	Takasugi et al., 2017
	Wnt10b	Induction of breast cancer metastasis to liver	Chen et al., 2017
	miR-21, −278e, and −143	Activation of EMT in breast cancer	Donnarumma et al., 2017
	miR-33a, −326	Influence in tumor cell adhesion, endocytosis and cell-cell junction	Nouraee et al., 2016
Metabolic Switch	miRNA- 302d, −29b, −22, −155, −25, −29a, −23a, −21, −16, −222, −24, −199a, −125b, −144	Down-regulation of genes related to OXPHOS by CAF exosomes	Zhao et al., 2016
	Lactate, Acetate, Citrate, Pyruvate, α-Ketoglutarate, Fumarate, Malate, aminoacids such as Gln, Arg, Glu, Pro, Ala, Thr, Ser, Asn, Val, Leu, Phe, Ile, Gly, or lipids such as Stearate and Palmitate	Modulation of glycolysis, tricarboxylic acid cycle, lipid and protein synthesis by CAF exosomes	Zhao et al., 2016
Chemoresistance	miR-146a, Snail	Induction of CAF chemoresistance	Richards et al., 2017

The genetic profile of fibroblasts that take up exosomes also interferes with the effectiveness of the cell-to-cell communication mediated by exosomes. For example, fibroblasts deficient in BRCA1, a tumor suppressor gene with role in DNA repair, internalize bigger amounts of serum-derived exosomes when compared to wild type counterparts (Hamam et al., 2016). In addition, these cells were shown to suffer malignant transformation when exposed to sera-derived exosomes from oncologic patients. It suggests that oncosuppressor genes can prevent integration of exosome information (including those from tumor cells) and protect these cells from pro-tumorigenic messages (Abdouh et al., 2017).

It has also been shown that tumor-derived exosomal effects in fibroblasts is also influenced by the aggressiveness of carcinoma cells. For example, when internalized by stromal fibroblasts, mammary carcinoma-derived exosomes carrying the Neuroblast Differentiation-associated protein AHNAK induce phenotype shifting of CAF, which is frequently associated with cancer progression. Notably, the expression of AHNAK varies according to the aggressiveness of the tumor, being low in benign epithelial breast cells, intermediate in localized malignant cells and high in metastatic cells (Silva et al., 2016).

Tumor-Fibroblast communication mediated by exosomes is not limited to local tissues, as it has also been described at distant tumor-associated microenvironments, such as pre-metastatic niches. For example, once internalized by fibroblasts, breast, and colorectal cancers and leukemia cells-derived exosomes containing the transcript of the enzyme Telomerase hTERT mRNA contributed to the establishment of pre-metastatic niches by increasing fibroblast proliferation and lifespan (Gutkin et al., 2016). In the context of malignant-ascites, gastric and ovarian tumor-derived exosomes may participate in the transformation of normal mesothelial cells into CAF by Mesothelial-Mesenchymal transition. Also, the augmented expression of CAF markers, such as Fibroblast Activation Protein, α-SMA and Fibronectin, can induce TGF-β1 expression, increasing peritoneum fibrosis and preparing this site for metastasis (Wei et al., 2017). Similar fibrotic effects have been found in exosomal CD151 and Tetraspanin 8 (Tspan8), which are essential components in the crosstalk between cancer initiating cells and their respective tumor-associated cells by, for instance, contributing to ECM remodeling (Yue et al., 2015). In addition, exosomes derived from lung-tropic tumors, such as some types of breast cancers, express high levels of Integrins α6β1 and/or α6β4, which allow them to specifically bind to lung fibroblasts, induce upregulation of S100A4, A6, A10, A11, A13, and A16, and lead to the formation of lung-pre-metastatic niches supportive of metastasis (Hoshino et al., 2015).

The crosstalk between stromal and tumor cells is bi-directional, and hence CAF-derived exosomes may act in tumor cells as well as in other non-tumor cells of the tumor microenvironment (Table 1). In esophageal cancer, for instance, it was shown that CAF-derived exosomes containing several microRNA species, including miR-33a, miR-326, play a role in tumor progression, influencing tumor cell adhesion, endocytosis, and cell-cell junctions (Nouraee et al., 2016). Furthermore, pancreatic CAFs, cells intrinsically resistant to the chemotherapeutic agent Gemcitabine, secrete chemoresistance-inducing exosomes when grown in the presence of this drug. This process, mediated by exosomal miR-146a and Snail, has been shown to mediate transfer of resistance to chemosensitive L3.6 and chemoresistant PANC1 and AsPC1 pancreatic cancer cells (Richards et al., 2017). Moreover, it was shown that inhibition of exosomes secretion by CAF prevented chemoresistance in cases of colorectal carcinoma (Hu et al., 2015). The growth of cancer cells can also be influenced by the altered secretome of senescent cells. In fact, exosomes from senescent fibroblasts induce proliferation of MCF-7 human breast cancer cells, in a mechanism mediated by EphA2 (Takasugi et al., 2017). CAF-derived exosomes are also capable to support tumor growth by providing nutrients to malignant cells. In fact, by modulating mitochondrial oxidative phosphorylation and glycolysis, CAF-derived exosomes can contain complete metabolites that may be used under nutrient deprivation stress conditions by tumor cells (Zhao et al., 2016).

Another important mediator in CAF differentiation and tumor biology is P85-α, as the downregulation of this factor promotes not only cancer progression via EMT by the Wnt10b paracrine pathway, but also metastatic progression. In addition, P85-α downregulation in fibroblasts is associated to Wnt10b upregulation in fibroblast-derived exosomes, which in turn induce breast cancer metastatic progression to liver (Chen et al., 2017). EMT is also modulated by CAF-derived exosomes containing miRs-21, −278e, and−143, influencing breast cancer cell phenotype and aggressiveness (Donnarumma et al., 2017). Taking advantage of this communication route involving modulation of tumor biology by fibroblast-derived exosomes, Kamerkar et al. showed that normal fibroblast-like mesenchymal cells can be engineered to carry interfering RNA or Short Hairpin RNA. Specifically, it was shown that engineered cells can produce exosomes capable to preferentially bind to tumor cells, target oncogenic KRAS and suppress cancer in multiple mouse models of pancreatic cancer (Kamerkar et al., 2017).

## Exosomes in tumor-endothelial cells communication

Endothelial cells (ECs) are key components of the tumor microenvironment not only by providing a conduit to nutrients, but also by representing a source of trophic factors. In this context, exosomes also play an instrumental role in tumor-EC communication (Table 2). For instance, the process of neovascularization was shown to be modulated by Myeloid leukemia-derived exosomes enriched in Vascular Cell Adhesion Molecule (VCAM)-1 and Intercellular Adhesion Molecule (ICAM)-1 (Taverna et al., 2012). In addition, increased vascularization has been associated with packaging of miR-92a in leukemia-derived exosomes (Umezu et al., 2013) and of CO-029/D6.1A Tetraspanin in pancreatic cancer-derived exosomes (Gesierich et al., 2006). It was also demonstrated that upregulation of Heparanase in tumor cells, including myeloma and breast cancers, is associated with increased exosomes production and exosomal packaging of Syndecan-1, Vascular Endothelial Growth Factor (VEGF) and Hepatocyte Growth Factor, which lead to increased endothelial invasion through the ECM (Thompson et al., 2013). Additionally, skin cancer-derived exosomes can promote angiogenesis by delivering Epidermal Growth Factor receptor (EGFR) (Al-Nedawi et al., 2009) and miR-9 to ECs (Gajos-Michniewicz et al., 2014). Furthermore, melanoma-derived exosomes were shown to condition sentinel lymph nodes prior to the installation of melanoma cells and further metastasis through upregulation of Collagen 18 and Laminin 5, and production of angiogenic growth factors (Hood et al., 2011).

**Table 2:** Effect of tumor-derived exosomes in endothelial cell (EC) biology.

**Process**	**Exosomal factor**	**Effect**	**References**
Angiogenesis	miR-92a and CO-029/D6.1A Tetraspanin	EC tube formation	Gesierich et al., 2006; Umezu et al., 2013
	Syndecan-1, Vascular Endothelial Growth Factor (VEGF), Hepatocyte Growth Factor	EC invasion	Thompson et al., 2013
	Epidermal Growth Factor Receptor (EGFR) and miR-9	Angiogenesis	Al-Nedawi et al., 2009; Gajos-Michniewicz et al., 2014
	MMP9, Pentraxin 3, IL-8, PDGF-AB/AA, CD26, Plasminogen Activator Inhibitor 1, Caveolin 1, Tissue Factor, Factor VIIa, Wnt4, miR-210, miR-23a	ECs proliferation and angiogenesis	Svensson et al., 2011; King et al., 2012; Kucharzewska et al., 2013; Huang and Feng, 2017; Sruthi et al., 2017
Integrity of vascular barriers	miR-105	Destruction of endothelial barriers and promotion of metastasis	Zhou W. et al., 2014
	miR-181c	Breakdown of blood-brain barrier	Tominaga et al., 2015
	VEGF-A	Increase in ECs permeability and angiogenesis	Treps et al., 2017

Tumor-derived exosomes are also known to influence the integrity of vascular barriers, which is frequently associated with metastatic dissemination. Melanoma-derived exosomes, for instance, induce pulmonary vascular leakiness (Peinado et al., 2012) and upregulate genes related to tumor cell recruitment, such as Stabilin 1, Vitronectin, Integrins, and Ephrin receptor β4, in lymph nodes (Hood et al., 2011), creating pre-metastatic niches supportive of metastasis. In addition, breast cancer-derived exosomes enriched in miR-105 targets ECs tight junctions by modifying the expression of Claudin 5, Zonula Occludens protein 1, and Occludin, promoting metastasis by destroying vascular endothelial barriers (Zhou M. et al., 2014). Another example involves brain tumor-derived exosomes containing miR-181c, which modulates ECs actin dynamics and promote breakdown of the blood-brain barrier by 3-Phosphoinostide-dependent Protein Kinase-1 degradation (Tominaga et al., 2015). Similarly, exosomes produced by glioblastoma cells containing high levels of VEGF-A induce ECs permeability and angiogenesis *in vitro* (Treps et al., 2017).

Hypoxia is another important factor in modulating tumor-EC communication. For example, hypoxic glioblastoma cells release exosomes that interact with ECs, stimulating proliferation and angiogenesis *in vitro* and *in vivo* (Skog et al., 2008; Kucharzewska et al., 2013), and also triggering Tissue Factor/Factor VIIa-dependent activation of hypoxic ECs (Milia et al., 2002; Svensson et al., 2011). Moreover, hypoxic colorectal cancer cells secrete Wnt4-enriched exosomes that promote Beta-Catenin nuclear translocation and proliferation of ECs (Huang and Feng, 2017), while hypoxic breast cancer and hepatocellular carcinoma cells were shown to release pro-angiogenic exosomes enriched in miR-210 (King et al., 2012) and miR-23a (Sruthi et al., 2017), respectively. In addition, exosomes produced by the human squamous carcinoma cell lineage A431 under hypoxic or reoxygenation conditions were shown to modulate the tumor microenvironment by facilitating angiogenesis, and as consequence, metastasis (Park et al., 2010).

## Exosomes in tumor-leukocytes communication

One of the hallmarks of cancer is the ability of tumor cells to employ different strategies to evade the host immune surveillance (Hanahan and Weinberg, 2011). Contemporary evidence points toward a central role of tumor-derived exosomes in modulating the immune response and influencing cancer development by mediating the dialogue between immune and cancer cells (Czernek and Düchler, 2016). In fact, tumor-derived exosomes have been shown to hijack the immune surveillance program of the host by amplifying tumor-derived signals, including those involved in inflammation, and in certain cases, tumorigenesis (Grivennikov et al., 2010; Cavallo et al., 2011) and escape of tumor cells from the immune system (Wieckowski et al., 2009; Whiteside, 2013).

**Figure 2:**
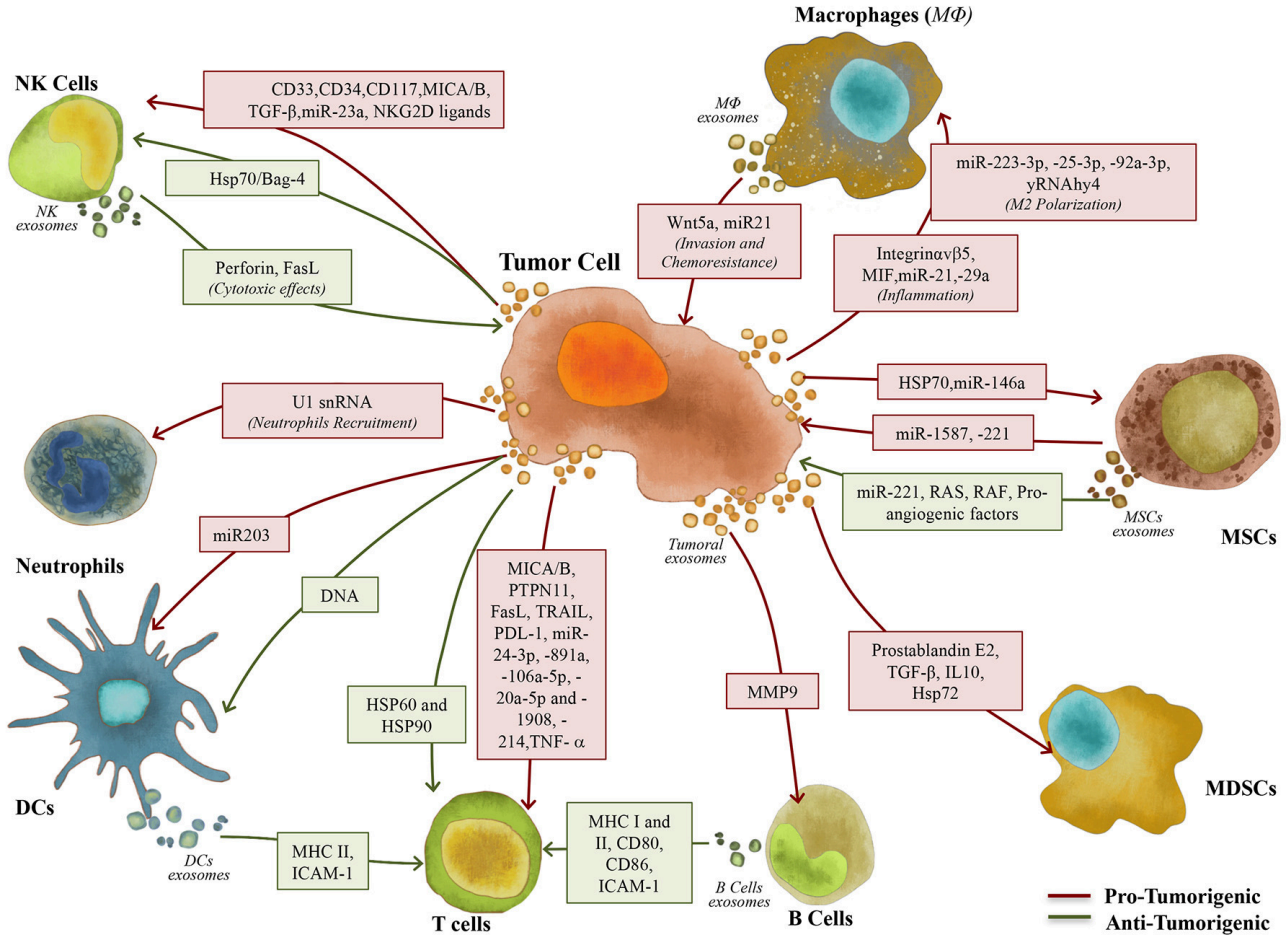
Exosomes role in the communication between tumor and immune/stem cells. Exosomes play a key role in the interaction between tumor and immune/stem cells, including Macrophages (MΦ), Natural Killer (NK) cells, Neutrophils, Dendritic Cells (DCs), B and T cells, Myeloid-Derived Suppressor Cells (MDSCs) and Mesenchymal Stem Cells (MSCs), contributing to the setup of pro- (red arrows) and anti-tumorigenic (green arrows) responses.

It is also known that tumor-derived exosomes are not only key players in the immune editing of the primary tumor niche, but also in the pre-metastatic and metastatic niches, as they can outsmart stromal and immune players into overcoming the immune response (Hanahan and Weinberg, 2011), fostering the setup of pro-metastatic microenvironments (Syn et al., 2016). In this section, we will dissect how exosomes can mediate cell-cell communication between the cellular components of the immune system and tumor cells and play a role in tumor biology (Figure 2).

### Exosomes roles in macrophages and neutrophils polarization

All types of immune cells are potentially sensitive to tumor-derived exosomes immunomodulation effects. However, exosomes can induce different levels of modification in each of these cells, as it is the case with macrophages. Activated macrophages, for instance, display a remarkable phenotypic plasticity according to environmental cues, being usually divided into a continuum of M1 and M2 functionally polarized states. In general, based on Th1/Th2 polarization, M1 macrophages are pro-inflammatory and tumoricidal (classically activated), while M2 are anti-inflammatory and tumor supportive (alternatively activated) (Martinez et al., 2008; Gautier et al., 2012; Xue et al., 2014; You et al., 2015). In a tumor microenvironment, macrophages can be educated into Tumor-Associated Macrophages (TAMs) displaying M2 characteristics, promoting angiogenesis and releasing pro-tumorigenic growth factors, chemokines and cytokines (Mantovani et al., 2004; Rogers and Holen, 2011; Quatromoni and Eruslanov, 2012; Schiavoni et al., 2013). In this setting, tumor-derived exosomes have been shown to play a key role in the polarization status of macrophages. For instance, colorectal cancer-derived exosomes induce pro-tumorigenic macrophage phenotypes by using cytoskeleton centric proteins as functional units. In fact, cytoskeleton rearrangement is a primordial characteristic of macrophage activation and maturation (Chen Z. et al., 2016). Another example involves epithelial ovarian cancer, where exosomes containing miR222-3p induced a shift in the activation status of macrophages into a M2 polarization, in a process mediated by down-regulation of the SOCS3/STAT3 pathway (Ying et al., 2016). Furthermore, miR-25-3p- and miR-92a-3p-rich exosomes from human liposarcoma cell lines were observed to induce IL-6 secretion by TAMs, leading to an increase in liposarcoma cellular proliferation (Casadei et al., 2017). In chronic lymphocytic leukemia (CLL), tumor-derived exosomes containing noncoding Y RNA hY4 were shown to induce CLL-associated phenotypes in recipient monocytes, including the release of cytokines, such as C-C motif chemokine ligand 2 (CCL2), CCL4, and IL-6, and the expression of PD-L1, suggesting a potential exosome-based mechanism of immune escape (Haderk et al., 2017). In addition to modulate macrophage polarization, tumor-derived exosomes were shown to influence macrophages migration. For example, Bandari et al. showed in a recent report that when exposed to commonly utilized anti-tumor agents, such as Bortezomib, Carfilzomib, or Melphalan, myeloma cells produce Heparanase-rich exosomes that induce migration and TNF-α secretion by macrophages (Bandari et al., 2018).

Tumor-derived exosomes are frequently related to NF-κB activation in macrophages and promotion of pro-tumorigenic microenvironments. For instance, gastric cancer-derived exosomes were shown to induce NF-κB activation in macrophages, leading to an increase in the expression of pro-inflammatory factors such as IL-6 and TNF-α, in turn promoting the proliferation of gastric cancer cells (Wu et al., 2016). Similar observations were obtained by Chow et al., who showed that breast cancer-derived exosomes also stimulate the NF-κB pathway in macrophages, leading to the secretion of the pro-inflammatory cytokines IL-6, TNF-α, GCSF, and CCL2 (Chow et al., 2014). Another example involves lung tumor-derived exosomes containing miR-21 and miR-29a. These exosomes were shown to bind to Toll-like receptor (TLR)7 and TLR8, leading to NF-κB activation and secretion of the pro-metastatic inflammatory cytokines TNF-α and IL-6. In turn, these cytokines were shown to induce the formation of a pulmonary microenvironment supportive of lung metastatic burden (Fabbri et al., 2012).

TAMs are also known to induce inflammatory responses, playing a key role in the setup of tumor microenvironments supportive of metastasis formation and progression. For example, once taken up by hepatic resident macrophages, in a mechanism mediated by exosomal Integrin αVβ5 (Hoshino et al., 2015), pancreatic cancer-derived exosomes containing high levels of Macrophage Migration Inhibitory Factor (MIF) (Costa-Silva et al., 2015) were shown to induce upregulation of secreted factors associated with liver fibrosis, such as TGFβ (Costa-Silva et al., 2015), and pro-inflammatory genes involved with metastasis, such as S100A8 and S100P (Lukanidin and Sleeman, 2012; Hoshino et al., 2015). In response to this inflammatory microenvironment, hepatic stellate cells shift into an activated state marked by upregulation of Fibronectin expression. This Fibronectin-rich microenvironment then promotes the accumulation of bone marrow-derived macrophages and the formation of a pre-metastatic microenvironment supportive of liver metastasis (Costa-Silva et al., 2015).

In addition to mediate immune responses, macrophages can also modulate tumor-derived exosomes biodistribution. Subcapsular sinus CD169^+^ macrophages, for example, form a physical layer that block tumor-derived exosomes' dissemination in spleen and lymph nodes (Saunderson et al., 2014). In agreement with this, high density of CD169^+^ macrophages in lymph nodes positively correlates with longer overall survival in patients with colorectal carcinoma (Ohnishi et al., 2013). Pucci et al., however, showed that this macrophage barrier can be disrupted by melanoma-derived exosomes, permitting the entrance of these exosomes in the lymph node cortex, where they interact with B cells and foster tumor progression by inducing autoantibodies production (Pucci et al., 2016).

Tumor-derived exosomes are not the only players in tumor-macrophage communication, as macrophages-derived exosomes can also exert effects in tumor cells biology. For instance, macrophages-derived exosomes can induce tumor invasion by transferring Wnt5a from macrophages to cancer cells, leading to the activation of the β-Catenin-independent Wnt signaling pathway, in a process that defines a new strategy of malignant invasion by breast cancer (Menck et al., 2013). Moreover, TAM-derived exosomes were shown to modulate chemoresistance. In fact, Zheng et al. showed that exosomes produced by TAM containing miR-21 could confer Cisplatin resistance to gastric cancer cells, in a process mediated by down-regulation of PTEN (Zheng et al., 2017). Wu and colleagues highlighted the relevant role of exosomes in the tumor microenvironment, unraveling an additional layer of complexity in the tumor-host communication network. In this work, it was shown that TAM-derived exosomes could suppress migration of endothelial cells by targeting the miR-146b-5p/ TRAF6/NF-kB/MMP2 pathway. Interestingly, ovarian cancer-derived exosomes were able to revert this inhibitory effect by transferring lncRNAs (Wu et al., 2017).

Neutrophils are also known as key mediators of innate immune response, as they are essential to protect the host against infection and to support tissue repair (Mayadas et al., 2014). Similar to macrophages, neutrophils also display phenotypic plasticity, which is influenced by different tumor-derived signals and that can exert pro- or anti-tumor effects. Indeed, tumors can manipulate neutrophils early in their differentiation process, creating a diverse repertoire of functional polarization states (Fridlender et al., 2009; Coffelt et al., 2016). In this setting, exosomes have been shown as emerging mediators of tumor-neutrophil interactions. For instance, breast cancer-derived exosomes can promote tumor growth by inducing bone marrow-derived neutrophils recruitment to tumor sites (Bobrie et al., 2012). Furthermore, neutrophils have emerged as crucial mediators in the pre-metastatic niche development (Wculek and Malanchi, 2015), as breast cancer-derived exosomal RNAs can activate host stromal TLR3, inducing neutrophil recruitment, which is critical for the setup of pre-metastatic niches (Kenific et al., 2016; Liu et al., 2016).

### Exosomes' role in suppression of natural killers' cytolytic response

Natural killer (NK) cells are another important component of the immune system, as these innate lymphoid cells are able to assemble a rapid cytotoxic activity (“ready to kill”), allowing them to control microbial infections and tumor progression in a process regulated by a balance of activating and inhibitory signals (Morvan and Lanier, 2015). Different reports state that the cytotoxicity of NK cells is significantly impaired in oncologic settings, in part due to the immune suppressive effect of tumor-derived exosomes. This immune suppressive effect has been associated with altered expression of NK cell activating surface receptors. For instance, exosomes derived from plasma of acute myelogenous leukemia (AML) patients, containing CD33, CD34, CD117, MICA/MICB, and TGF-β, were shown to decrease cytotoxic activity of NK cells, in a process involving Smad phosphorylation and down-regulation of NKG2D receptor in NK cells (Szczepanski et al., 2011; Whiteside, 2013), which was reversible by IL-15 (Szczepanski et al., 2011). Another example involves the work of Berchem et al., who showed that tumor-derived exosomes produced by hypoxic cells are qualitatively different from normoxic counterparts. Using various tumor models, they revealed that hypoxic tumor-derived exosomes were able to negatively regulate NK cell functions, in a mechanism also mediated by transfer of TGF-β1 and decrease of NKG2D levels in NK cells. The authors also described miR-23a and TGF-β as an immunosuppressive factor transferred to NK cells that directly targets CD107a expression, leading to a decrease of anti-tumor responses (Berchem et al., 2016). In fact, in the presence of tumor-derived exosomes, NKG2D is one of the most profoundly affected NK receptors, which have MHC class I-related chain A (MICA) and B (MICB) as crucial ligands for its induction (Mincheva-Nilsson and Baranov, 2014). Accordingly, Groh et al. showed that cancer patients exhibit a decrease of NKG2D surface expression on circulating NK cells in comparison with healthy individuals (Groh et al., 2002). Furthermore, Ashiru et al. demonstrated that the shedding of the most frequently expressed MICA allele in human populations, MICA^*^008, in tumor-derived exosomes induce down-regulation of NKG2D in NK's cell surface, leading to an impaired cytotoxic function (Ashiru et al., 2010). Moreover, a recent study showed that prostate tumor-derived exosomes with NKG2D ligands selectively induce a dose-dependent downregulation of cell surface NKG2D in both NK cells and CD8+ T cells, leading to impaired cytotoxic function of both immune cell types (Lundholm et al., 2014). Tumor-derived exomes bearing NKG2D ligands (such as MIC-A/B and ULBP 1 and 2) were also shown to act as decoys, impairing NKG2D-mediated NK-cell cytotoxicity and facilitating the immune evasion of leukemia/lymphoma cells (Hedlund et al., 2011).

An additional mechanism of immune evasion was described by Liu et al., who showed that murine mammary-derived exosomes were able to inhibit IL-2-stimulated NK cells proliferation and block IL-2-mediated activation of NK cells, thus abolishing their cytotoxic response to tumor cells (Liu et al., 2006). In addition, tumor-derived exosomes containing death receptor ligands, such as FasL, on their membrane were also found to induce the apoptosis of NK cells, similarly to what happens with T cells (Andreola et al., 2002; Saito et al., 2013). On the other hand, anticancer drugs have been described to induce chemoresistant hepatocellular carcinoma cells to release exosomes that elicit anti-tumor NK cell responses, in a mechanism mediated by exosomal Heat Shock Proteins (Lv et al., 2012). Similar to other tumor-immune cell-cell communication settings, NK cells have also been shown to influence tumor cells biology though production of exosomes. It was illustrated in a recent work of Zhu et al., showing that NK cells-derived exosomes can induce cytotoxic effects in melanoma cells *in vitro*, representing a potentially novel antitumor strategy (Zhu et al., 2017).

### Exosomes role in impairment of cytotoxic lymphocytes (CTL) response and induction of immune tolerance/immune regulator cells

The centerpiece of antitumor immunity is the effective response of CD4^+^ and CD8^+^T cells. In this setting, tumor-derived exosomes have been also described as potent mediators of proliferation, activation, and apoptosis of these cells, enabling tumor evasion from immune surveillance. For instance, T cell death has been shown to be induced by tumor-derived exosomes through both extrinsic and intrinsic apoptotic pathways. In fact, induction of T cells apoptosis by tumor-derived exosomes occurs through receptor-mediated pathways involving Fas Ligand (FasL), TRAIL, and PDL-1 (Abusamra et al., 2005; Kim et al., 2005; Wieckowski et al., 2009). In addition, Abusamra et al. demonstrated that prostate cancer-derived exosomes expressing FasL trigger T-cell apoptosis in a dose-dependent fashion (Abusamra et al., 2005). In another example, head and neck squamous cell carcinoma-derived exosomes were shown to induce activated CD8^+^T cells apoptosis through Caspase 3 cleavage, mitochondrial Cytochrome C release, loss of mitochondrial membrane potential, DNA fragmentation and early membrane changes, including Annexin V binding (Czystowska et al., 2011). The PI3K/AKT pathway is also targeted by exosomes in activated CD8^+^ T cells, as tumor-derived exosomes were shown to cause Akt dephosphorylation in a time-dependent manner, leading to downregulation of the anti-apoptotic proteins Bcl-2, Bcl-xL, and Mcl-1 and upregulation of the pro-apoptotic protein Bax (Czystowska et al., 2009, 2011). Furthermore, the production of tumor-derived exosomes containing MICA/B and FasL is associated with impairment of the effectives of both innate and adaptive immunity (Abusamra et al., 2005; Lundholm et al., 2014). Therefore, it has been suggested that such exosomes are able to modulate lymphocyte functions by mimicking “activation-induced cell death” (AICD) (Blanchard et al., 2002; Perone et al., 2006; Taylor and Gercel-taylor, 2011). Another example involves melanoma-derived exosomes, which were shown to suppress CD8^+^T cell proliferation and viability by delivering PTPN11 (protein and mRNA) (Wu et al., 2017). In addition, human nasopharyngeal carcinoma-derived exosomes can inhibit T cell proliferation and differentiation into Th1 and Th17 cells, promoting regulatory T cell (Treg) generation, decreasing ERK, STAT1, and STAT3 phosphorylation and increasing STAT5 phosphorylation in the recipient T cells. These tumor-derived exosomes also increased the secretion of the pro-inflammatory cytokines IL-1β, IL-6, and IL-10, and decreased the release of IFNγ, IL-2, and IL-17, both in CD4^+^ and CD8^+^T cells. The content of exosomes from patient sera and nasopharyngeal carcinoma cell lines was further explored, showing to be enriched in miR-24-3p, −891a, −106a-5p, −20a-5p, and−1908. The same work showed that these miRNA clusters downregulate the MARK1 signaling pathway, affecting T cells proliferation and differentiation (Ye et al., 2014).

Unresponsiveness of CD8^+^ T cells can also be achieved by damaging the TCR (T-cell receptor) signaling pathway, which mediate TCR–MHC–peptide interactions and T cell activation, in a mechanism involving CD3ζ chain transfer of activating signals to the nucleus. Soderberg et al. demonstrated that melanoma-derived exosomes transfer TNF-α to CD4^+^ and CD8^+^T cells, affecting the TCR–CD3 complex, and causing T cell disruption (Soderberg et al., 2007). In agreement, several studies also reported that tumor-derived exosomes from cancer patients mediate inhibition of CD3ζ chain expression in T cells, impairing T cell activation (Taylor et al., 2003; Kim et al., 2005; Taylor and Gercel-taylor, 2011).

Another critical step in the anti-tumor immune response is the CD4^+^ and CD8^+^T cells homing to the primary tumor site (Taylor et al., 2003; Kim et al., 2005; Taylor and Gercel-taylor, 2011). Lee et al. showed that by shedding ICAM1 in the membrane of their exosomes, cancer cells prevent the interaction between lymphocytes and endothelial cells, thus decreasing the recruitment of adaptive immune cells (Lee et al., 2010).

Besides impairment of CTL responses, another mechanism present in tumor cells which promote escape from immune surveillance involves interference in the differentiation process of naïve immune cells toward an immunosuppressive phenotype. For example, tumor-derived exosomes are capable of inducing expansion of CD4^+^CD25^+^Foxp3^+^ Treg cells and their suppressor activity, and to trigger CD8^+^T cells apoptosis (Wieckowski et al., 2009). Additionally, exosomes produced by various types of human and murine tumors were reported to transfer miR214 to peripheral CD4^+^ T cells, stimulating immune evasion by downregulating PTEN and promoting Treg expansion (Yin et al., 2014).

In a recent study, Muller et al. showed that tumor-derived exosomes can also induce differential regulation of key immune function-related genes in conventional CD4^+^ T, CD8^+^ T, and Treg. In CD4^+^ T cells, for instance, inhibitory genes were upregulated by tumor-derived exosomes, which led into a loss of CD69 on their surface and a functional decline of these cells. On the other hand, exosomes induced CD39 expression and adenosine production in Treg, while downregulating mRNA expression levels of the genes involved in the control of immmunosuppressive pathways (Muller et al., 2016). Collectively, by exploiting diverse mechanisms acting in concert, tumor-derived exosomes contribute to an immunosuppressive environment and peripheral tolerance, which play pivotal role in tumor growth and progression.

### Exosomes role in DCs differentiation and maturation

Dendritic cells (DCs) are versatile meditators of the immune system, forming a remarkable network that shapes innate and adaptive immunity, according to peripheral signals (Merad et al., 2013). The DCs importance is associated with their function as professional antigen-presenting cells (APCs), since they are able to perform antigen presentation and initiation of primary T cell response, including those frequently directed against tumor cells (Liu et al., 2017).

The presence of tumor-derived exosomes during the generation of DCs was associated with low expression of co-stimulatory molecules and production of inhibitory cytokines by these cells, in a process followed by suppression of T cells proliferation and anti-tumor cytotoxicity (Valenti et al., 2006). In addition, tumor-derived exosomes taken up by immature DCs were shown to inhibit the maturation process of these cells (Yang et al., 2011).

The antigen recognition capacity of DCs can also be modulated by tumor-derived exosomes, which affect the expression of pattern recognition receptors (PRRs) in DCs. Pancreatic cancer-derived exosomes, for instance, can downregulate the expression of TLR4 (via miR-203) in DCs, inducing the production of TNF-α and IL-12 and inhibiting DCs-mediated antitumor responses triggered by TLR4 (Zhou M. et al., 2014). In summary, increasing number of reports have been showing that tumor-derived exosomes can also mediate host immunosuppression by modulating the differentiation, maturation and function of DCs.

### Exosomes role in B cells

B cells are important players in the tumor-induced modulation of immune response, being the second most abundant tumor-infiltrating lymphocytes (Yuen et al., 2016). In 1996, Raposo et al. reported for the first time that B-lymphocytes secrete exosomes, and that these particles contain MHC class II able to perform antigen presentation and to induce antigen-specific CD4+ T cell responses (Raposo et al., 1996). In addition to the several reports of exosomes production by B cells, and of the potential roles of these particles in the immune system (Escola et al., 1998; Clayton et al., 2005; Rialland et al., 2006; Buschow et al., 2010), these cells were also reported to be influenced by tumor-derived exosomes. In fact, esophageal cancer-derived exosomes containing HSP90 can influence naïve B cells to develop into an immunosupressive regulatory phenotype expressing TGF-β (Li Y. et al., 2015). Furthermore, melanoma and lymphoma cells were shown to release exosomes that induce IL-10 production in splenic B cells. In turn, IL-10 promote the generation of regulatory B cells which inhibit T cell activity (Yang et al., 2012), suggesting a novel mechanism of induction of inhibitory B cells based on exosome-driven pathways.

### Exosomes involvement in the stimulation of immunity against cancer

Exosomes produced by cancer cells are known to predominantly induce immune suppression and to support tumorigenesis. However, several reports show that these nanoparticles are also capable of stimulating immunity (Reiners et al., 2014; Czernek and Düchler, 2016; Liu et al., 2017; Yoshimura et al., 2017). This dual role of tumor-derived exosomes is mainly due to their ability to express tumor-associated antigens. In addition, exosomes are considered ideal sources of antigens for APCs education, especially due to its easy collection from the peripheral circulation by the use non-invasive methods. In fact, early reports showed that antigens in tumor-derived exosomes can be transferred to DCs and induce specific anti-tumor immune responses (Andre et al., 2002; Robbins and Morelli, 2014). For instance, DCs pulsed with hepatocellular carcinoma cell (HCC)-derived exosomes led to an increase in IFNγ levels and CD8+ T cells, and in the reduction of IL10 and TGF-β levels in HCC-bearing mice (Rao et al., 2016). Another example involves cancer cells treated with anti-tumor agents, which can release DNA-containing exosomes capable of activating DCs though the STING-dependent pathway and reinforce antitumor immunity (Kitai et al., 2017).

Additionally, tumor-derived exosomes can enhance anti-tumor immunity through transfer of cytokines or heat shock proteins (Liu et al., 2017). In fact, HSP60 and HSP90 are abundant in exosomes derived from heat-shocked mouse B lymphoma cells, being these exosomes associated with increased antitumor immune responses by T cells (Chen et al., 2006). In line with these findings, tumor-derived HSP70/Bag-4+ exosomes stimulate NK cell activity, inducing granzyme B release and pancreatic cancer cell apoptosis (Gastpar et al., 2005). Based on these anti-tumor effects generated by tumor-derived exosomes, several lines of research have been focusing in developing exosome-based tumor vaccines. Common strategies involve genetic modification of exosomes-producing cells in order to modify exosomes content, including IL2 (Yang et al., 2007) and IL18 (Dai et al., 2008) and improve exosome-driven immunogenicity. In addition, other strategies are based on stimulating the release of exosomes that act on NK cell toxicity by stressing tumor cells with, for instance, anti-cancer drugs (Lv et al., 2012).

The ability to induce effective immunity is not limited to tumor-derived exosomes, as immune cells can also release exosomes able to act on the immune system and elicit antitumor responses. DC-derived exosomes, for instance, can act as antigen-presenting particles (Zitvogel et al., 1998) and stimulate antigen-specific cytotoxic T lymphocytes *in vivo* (Raposo et al., 1996; Zitvogel et al., 1998). In addition, DC-derived exosomes containing ICAM-1 and B cells-derived exosomes containing MHC Class I and II molecules, co-stimulatory molecules (CD80 and CD86) and ICAM-1 were shown to promote antigen-presenting function in recipient immune cells (Clayton et al., 2001; Théry et al., 2001; André et al., 2004; Segura et al., 2005). These exosomes can modulate immune responses either directly, by exposing these molecules, or indirectly, by conveying internal components to surrounding cells. Taken the effects mentioned above, DC-derived exosomes represent an important strategy to suppress tumor growth through novel cell-free vaccination approaches (Tian and Li, 2017).

To date, DC-based cellular immunotherapy has exhibited several limitations, such as fluctuations in the molecular composition of DCs, challenges in defining its composition, low levels of membrane expression of peptide-MHC-II complexes, presence of immunosuppressive cytokines able to convert DCs into tolerogenic state, hindrances to store live DCs and problems regarding stability management over longer periods (Palucka and Banchereau, 2012; Pitt et al., 2016). When compared to DC-based cellular immunotherapy protocols, DC exosomes-based strategies present significant advantages, as exosomes have better defined molecular composition for each patient, higher levels of peptide–MHC-II complexes and higher stability for storage due to their lipid composition (Pitt et al., 2016; Yoshimura et al., 2017). Although further studies are still necessary, important progress has been made in clinical applications of DC exosomes-based vaccines. Indeed, patients with advanced malignancies, including those with non-small cell lung cancer (Morse et al., 2005; Besse et al., 2016), metastatic melanoma (Escudier et al., 2005) and colorectal cancer (Dai et al., 2008), that were vaccinated with DC-derived exosomes displayed activation of T and NK cell-based immune responses. In addition, two phase I (Escudier et al., 2005; Morse et al., 2005) and one phase II clinical trial (Besse et al., 2016) using DC-derived exosomes have now been completed, showing the feasibility and safety of this approach.

## Exosomes in tumor-stem/progenitor/non-transformed cell communication

Besides the well-known effects in differentiated cells, tumor-derived exosomes can also induce pro-tumorigenic microenvironments by mediating tumor-stem/progenitor cell communication. Melanoma-derived exosomes, for instance, “educate” bone marrow-derived cells via the horizontal transference of the oncoprotein MET, which leads to mobilization of vasculogenic and hematopoietic bone marrow progenitor cells to ensure vascular proliferation and immunosuppression at the pre-metastatic niches (Peinado et al., 2012). Tumor-stem/progenitor cell communication was also described in scenarios of bone metastasis, as exosomes from the bone-metastatic prostate cancer PC3 cells were shown to modulate both osteoclastogenesis and osteoblast proliferation, influencing the process of bone metastasis. In turn, osteoblast-derived exosomes were shown to promote PC3 prostate cancer cell proliferation (Morhayim et al., 2015).

In addition, tumor-derived exosomes were shown to manipulate the process of myeloid precursor cells differentiation toward myeloid-derived suppressor cells (MDSCs), which are known to contribute to tumor progression by allowing immune evasion (Nagaraj and Gabrilovich, 2007). Exosomes derived from breast carcinomas, for instance, were shown to be taken up by bone marrow cells and to switch the differentiation pathways of these cells toward MDSCs via Prostaglandin E2 and TGF-β, promoting accumulation of COX2, IL6, VEGF and Arginase1 by MDSCs (Xiang et al., 2009). Similarly, glioma stem cell-derived exosomes were shown to act on MDSCs via IL10 and Arginase 1 upregulation and HLA-DR downregulation in CD14+ monocytic MDSCs, leading to inhibition of T cell activation (via CD25 and CD69) and decrease of Th1 cytokine production (Domenis et al., 2017). In agreement with these findings, tumor-derived exosomes prevented the process of monocytes maturation by switching differentiation toward altered CD14^+^ monocytes expressing low or absent levels of HLA-DR, which was shown to suppress T cell proliferation and cytolytic functions (Valenti et al., 2006; Temme et al., 2010). Additionally, Chalmin et al. showed that HSP72+ exosomes derived from several tumor models can stimulate the suppressive function of MDSCs via Stat3 activation, leading to IL-6 production in a MyD88/TLR2-dependant mechanism (Chalmin et al., 2010). This finding was reinforced by the involvement of MyD88, a molecule involved in the propagation of signals generated by the TLR family and production of IL-6, TNF-α, and CCL2, in the recruitment and activity of MDSCs by tumor-derived exosomes (Liu et al., 2010). In another example, Wang and colleagues described that multiple myeloma-derived exosomes can create a pro-tumorigenic microenvironment in the bone marrow by inducing MDSCs expansion and activity, endothelial cell growth via the STAT3/p53 pathway and immunosuppression via upregulation of Nitric Oxide Synthase (Wang et al., 2016). Finally, melanoma-derived exosomes can prevent bone marrow myeloid precursors differentiation into DCs via induction of IL-6, favoring the MDSC phenotype (Valenti et al., 2006).

Cancer-derived exosomes can also elicit changes in mesenchymal stem cells (MSCs), which contribute to promote and sustain pro-tumorigenic inflammatory niches. For instance, HSP70^+^ lung tumor-derived exosomes induce activation of NF-kB and secretion of IL-6, IL-8, and MCP1 by MSCs, in a TLR2-mediated signaling, leading MSCs into a more inflammatory and tumor supportive phenotype (Li et al., 2016). In a recent report, De Veirman et al. showed that myeloma-derived exosomes can transfer miR-146a to mesenchymal cells, leading to the secretion of several cytokines and chemokines by these recipient cells, including CXCL1, IL6, IL-8, IP-10, MCP-1, and CCL-5, and to the promotion of myeloma cells migration and survival (De Veirman et al., 2016). Another example involves exosomes produced by KMBC cholangiocarcinoma cells that induce IL-6 upregulation in MSCs, which in turn promotes the proliferation of KMBC cells (Haga et al., 2015). In addition, ovarian and breast cancer-derived exosomes can induce MSCs differentiation into CAFs, resulting in increased metalloproteinase activity and expression of a vast array of factors involved in the remodeling of epithelial adherent junction pathways, thus enhancing tumor cell invasiveness and aggressiveness (Clayton et al., 2007; Cho et al., 2011a,b). Similarly, prostate cancer-derived exosomes can induce a process of tumor mimicry through the expression of epithelial neoplastic and vascular markers by adipose tissue-derived MSC, together with the acquisition of aberrant cytogenetic variations and Mesenchymal-to-Epithelial transition (Abd Elmageed et al., 2014). Moreover, breast cancer-derived exosomes also act on adipose tissue-derived MSCs, inducing the secretion of SDF-1, VEGF, CCL5 and TGF-β, which was associated with differentiation of these MSCs into tumor-associated myofibroblasts (Cho et al., 2011a).

Like other exosomes-based tumor-stroma interaction circuits, tumor-MSCs communication is also bidirectional. For instance, exosomes released by MSCs can upregulate the expression of VEGF in tumor cells via activation of the ERK1/2 and p83 mitogen-activated protein kinase pathways, increasing ECs proliferation and angiogenesis, therefore supporting tumor growth (Zhu et al., 2012). Another example involves exosomes enriched in miR-1587 produced by glioblastoma-associated MSC, which were shown to increase the proliferation and clonogenicity of tumor-initiating glioma stem-like cells (Figueroa et al., 2017). Interestingly, bone marrow, umbilical cord, and adipose tissue MSC-derived exosomes displayed differential effects on the proliferation of U87MG glioblastoma cells, indicating that the origin of the stem cells might be of relevance toward its pro-tumorigenic effects (Del Fattore et al., 2015). Furthermore, exosomes produced by bone marrow-derived MSC containing high levels of miR-221 induced proliferation, migration, invasion and adhesion of gastric cancer cells. In addition, exosomal expression of miR-221 was also associated with poor prognosis in patients with gastric cancer (Ma et al., 2017).

Another relevant aspect influenced by exosomal tumor-stem/progenitor cell communication is chemoresistance. Although chemoresistance frequently originates from selection of resistant tumor cell clones after drug exposure, drug resistance can also rise from the transference of biomolecules produced by stromal cells to exosomes. In pre-clinical models, for example, bone marrow stromal cell-derived exosomes induced tumor migration, proliferation and drug resistance by reducing the expression of apoptosis-related proteins by multiple myeloma cells (Wang et al., 2014). In addition, MSC-derived exosomes were found to induce resistance to 5-Fluouracyl and Cisplatin via activation of the RAS/RAF/MEK/ERK pathway in gastric cancer cells (Ji et al., 2015). And, finally, exosomal RNA produced by stromal cells were shown to activate RIG-I anti-viral signaling in breast cancer cells, leading to the expansion of therapy resistant breast cancer cells in a mechanism involving NOTCH3 induction (Boelens et al., 2014).

Tumor-derived exosomes have also been shown to act in non-transformed differentiated cells counterparts, inducing the expression of malignant features by these recipient cells. For instance, Chen et al. showed that exosomes are able to mediate intercellular communication between neoplastic and normal cells, promoting a pro-inflammatory phenotype in the latter. Specifically, it was shown that exosomes from arsenite-transformed liver cells activate the IL6, IL8/STAT3 pathway, enhancing miR155 expression and inflammatory properties in normal liver cells (Chen C. et al., 2016). Moreover, exosomes derived from the MatLyLu metastatic prostate tumor cells were capable to induce higher levels of proliferation in normal prostate epithelium cells than exosomes from the less aggressive non-metastatic Dunning G prostate tumor cells (Halin Bergstrom et al., 2016).

## Summary

Exosomes are biological active vesicles and professional carriers of information in intercellular communication. In an oncologic scenario, they play a pivotal role in shaping the tumor microenvironment by affecting tumor growth and proliferation, mediating the crosstalk between tumor and tumor-associated cells and molding the host immune response. As emerging components in tumor-host crosstalk, exosomes modulate elementary steps of tumor progression, such as growth, invasion and immunosurveillance. In addition to effects in local tumor microenvironments, exosomes released from tumors were shown to mediate distant cell-cell communication processes which result in the setup of pro-tumorigenic microenvironments supportive of metastatic dissemination. This is achieved, for instance, though modulation of fibroblast activation, ECM production, angiogenesis and immunesurveillance. As both tumor- and stroma-derived exosomes are found abundantly at the peripheral circulation, they represent a precious opportunity to access non-invasively and in real time the biological status of the tumor microenvironment. Taking into account the relevance of non-tumor cells in cancer progression, this may provide a new source of biological markers with application in not only prediction of prognosis, but also in disease follow-up during and after therapeutic intervention. In addition to the current efforts in investigating the application of exosomes as anti-tumor tools (such as DC-derived exosomes), the further comprehension of the basic biology of exosomes, including the identification of exosomal components relevant for tumor progression, may represent an opportunity for novel therapeutic strategies based on the targeting of pro-tumorigenic exosomes-based cell-cell communication. However, in spite of the substantial expansion of the field, especially in the last decade, the roadmap to understand the exosomes communication system with the organism is still far from being fully understood. Besides the need for more information on fundamental issues, such as exosomes biogenesis and the mechanisms of exosomal cargo delivery, further effort is still necessary in the standardization of methods involving exosomes purification and characterization. The establishment of standard methods for exosomes isolation, as well as of a consensus regarding biological and physical characteristics that define a group of extracellular vesicles as exosomes, such as the one proposed by the EV-TRACK Consortium (EV-TRACK Consortium et al., 2017), are essential. This is of great relevance, as the myriad of methods for exosomes isolation described in the literature (such as ultracentrifugation, immuneprecipitation and size exclusion chromatography) has the potential to complicate the reproducibility of current and future works and the overall establishment and progress of this flourishing research field.

## Author contributions

BC-S: organization of content and structure, writing and reviewing; SC: writing exosomes role in tumor-fibroblast and tumor-endothelial cells communication, figures preparation; JM: writing exosomes role in tumor-immune system communication, preparation of figures; NC: writing exosomes role in tumor stem/progenitor cells communication. MS: writing introduction and the role of exosomes in tumor-tumor communication.

### Conflict of interest statement

The authors declare that the research was conducted in the absence of any commercial or financial relationships that could be construed as a potential conflict of interest.
